# TRIM66 promotes malignant progression of prostate carcinoma through the JAK/STAT pathway

**DOI:** 10.1002/2211-5463.12798

**Published:** 2020-03-03

**Authors:** Hongwen Cao, Renjie Gao, Lei Chen, Yigeng Feng

**Affiliations:** ^1^ Surgical Department I (Urology Department) LongHua Hospital Shanghai University of Traditional Chinese Medicine China

**Keywords:** IL‐2, prostate cancer, STAT2, TRIM66

## Abstract

Prostate cancer is the fifth leading cause of cancer‐related deaths in males globally. Tripartite Motif Containing 66 (TRIM66) functions as transcriptional repressor and exerts its effect at least partially through promotion of deacetylase. TRIM66 has been previously reported to play an oncogenic role in a number of human cancers. Here, we investigated the potential oncogenic properties of TRIM66 in prostate cancer. We report that shRNA‐mediated knockdown of TRIM66 significantly suppressed viability and proliferation of both PC‐3 and DU145 prostate cancer cell lines. Furthermore, TRIM66 deficiency inhibited migration and invasion of prostate cancer cells. Mechanistically, TRIM66 positively regulated signal transducer and activator of transcription 2 (STAT2) and interleukin‐2 (IL‐2) expression. The predominance of STAT2–IL‐2 in mediating the oncogenic properties of TRIM66 was determined using a rescue assay, wherein overexpression of either STAT2 or IL‐2 almost completely abolished the inhibitory effects on cell proliferation, migration and invasion elicited by TRIM66 deficiency in prostate cancer cells. Our study highlights the importance of the TRIM66–STAT2–IL‐2 signaling axis in the tumor biology of prostate cancer.

## Graphical abstract

Tripartite Motif Containing 66 (TRIM66) knockdown suppressed viability and proliferation of prostate cancer cell lines. TRIM66 deficiency inhibited migration and invasion of prostate cancer cells. TRIM66 positively regulated signal transducer and activator of transcription 2 and interleukin‐2 expression. Our study highlights the importance of the TRIM66–signal transducer and activator of transcription 2–interleukin‐2 signaling axis in the tumor biology of prostate cancer.

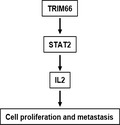

**Abbreviations**
CCK‐8: Cell Counting Kit‐8
EMT: epithelial–mesenchymal transition
IL‐2: interleukin‐2
SD: standard deviation
STAT2: signal transducer and activator of transcription 2
TCGA: The Cancer Genome Atlas
TRIM66: Tripartite Motif Containing 66

Prostate cancer is the second most common form of cancer and the fifth leading cause of cancer‐related deaths in males globally 1. It is estimated that 1.1 million new cases were diagnosed and 307 000 deaths were claimed by this disease according to Cancer Statistics 2012 2. The incidence of prostate cancer has been increasing in the last decade in developing countries, primarily because of the progression in prostate‐specific antigen testing, despite its controversial clinical benefits regarding endpoint outcomes 3. Acknowledged risk factors associated with tumorigenesis and progression of prostate cancer include age, family history and race 4. Dietary habits, such as excessive consumption of processed meat, red meat or milk products, were reported to be linked with etiology of this disease 5. In addition, genetic aberrances, especially mutations in the *BRCA* gene, are characterized to be intimately associated with initiation of prostate cancer 6.

Clinically, prostate cancer is frequently diagnosed and confirmed by biopsy, followed by medical imaging to evaluate the extent of potential metastasis. Management options for this disease greatly depend on tumor stage and general personal health conditions, and mainly consist of regular surveillance and combination of surgery, radiotherapy, chemotherapy and hormone therapy 7. The prognosis of prostate cancer is relatively favorable, and the 5‐year survival rate in the United States is more than 99%. Notwithstanding, insightful understanding into the molecular mechanisms underlying this disease is still in urgent need for better therapeutic purpose.

Tripartite Motif Containing 66 (TRIM66) belongs to the superfamily of Tripartite Motif Containing proteins, with potential functions as transcriptional repressor, which is at least partially mediated by recruitment of the deacetylase activity. TRIM66 is also speculated to function as a negative regulator of postmeiotic genes via CBX3 complex formation and centromere association. Several investigations indicate the oncogenic roles of TRIM66 in a number of human cancers. Chen *et al.* 8 first identified that overexpression of TRIM66 contributed to osteosarcoma incidence and was associated with poorer survival prediction. The study performed by Dai *et al.* 9 demonstrated that TRIM66 knockdown inhibited malignant behaviors and epithelial–mesenchymal transition (EMT) in non‐small cell lung cancer. Subsequently, Wang *et al.* 10 disclosed that long noncoding RNA TATDN1 contributed to the resistance to cisplatin in non‐small lung cancer via the miR‐451/TRIM66 signaling pathway. In hepatocellular carcinoma, Zhang *et al.* suggested that aberrantly overexpressed TRIM66 conferred transformation potential through regulating the GSK‐3β‐dependent Wnt/β‐catenin pathway 11.

However, the possible involvement and potential mechanistic contribution of TRIM66 in prostate cancer are currently elusive and yet to be comprehensively defined. Here we set out to address this issue and characterize the importance of TRIM66 in proliferation, cell cycle, migration and invasion of prostate cancer cells. Further mechanistic elucidation clearly uncovered the TRIM66–signal transducer and activator of transcription 2 (STAT2)–interleukin (IL)‐2 signaling axis in the malignant progression of this disease. Our study conferred insightful understanding into the fundamental molecular signaling cues, which definitely will benefit the clinical exploitation as therapeutic targets.

## Materials and methods

### Cell culture

Human prostate cancer cell lines PC‐3 and DU145 were purchased from and authenticated by the American Type Culture Collection (ATCC, Manassas, VA, USA). Cell identities were authenticated by Short Tandem Repeat profiling. Cells were maintained in Dulbecco’s modified Eagle’s medium containing 10% FBS (Hyclone, Waltham, MA, USA) and cultured in humidified CO_2_ incubator at 37 °C. The potential mycoplasma contamination was regularly examined by PCR method.

### Tissue samples

The prostate tumor paired with adjacent normal tissues was collected from LongHua Hospital Shanghai University of Traditional Chinese Medicine. The written informed consents were obtained from all patients enrolled in this study. Human‐related study conformed to the guidelines of the Declaration of Helsinki and was approved by the Institutional Committee of Ethics of LongHua Hospital.

### Real‐time quantitative PCR

Total RNA was isolated from indicated cell lines and tissues samples using TRIzol (Invitrogen, Waltham, MA, USA) and subjected to cDNA preparation with Transcriptor First Strand cDNA Synthesis Kit (Roche, Basel, Switzerland). The TaqMan gene expression assays were used to quantify relative expression following the manufacturer’s manual (Applied Biosystems, Foster City, CA, USA) on ABI HT‐7900 System (Applied Biosystems).

### Cell Counting Kit‐8 assay

The viability of indicated cells was determined using Cell Counting Kit‐8 (CCK‐8; Dojindo Laboratories, Kumamoto, Japan). Cells (4 × 10^3^ cells per well) were seeded in 96‐well plates and transfected with the desired shRNA followed by 48‐h incubation. At the end, 10 μL CCK‐8 working solution was added to each well and continuously incubated at 37 °C for 2 h. The viable cells were assessed by readout of *D*_450 nm_ with Multiscan MS (Labsystems, Hamburg, Germany).

### Direct cell counting

The indicated cells were consecutively cultured for 48 h and prepared into single‐cell suspension in PBS. Cell concentration was determined with hemocytometer, and dead cells were excluded by trypan blue staining.

### Cell migration and invasion assay

Migration of specified cells was assayed with Transwell chamber (8‐μm pore size; Corning, Corning, NY, USA). In brief, cells were digested with trypsin to single‐cell suspension in serum‐free medium and immediately added into the upper compartment. The lower chamber was supplemented with complete culture medium supplemented with 10% serum. After 12 h, the nonmigrating cells were cautiously removed with cotton swabs, and invaded cells were fixed with 4% paraformaldehyde and stained with 0.25% crystal violet for visualization and counting under light microscope. For invasion evaluation, the Transwell chamber was precoated with 1% Matrigel (Millipore, Billerica, MO, USA).

### Western blot

Protein was extracted by lysing cells in sample buffer, and an equal amount of protein (20 μg) was subjected to 10% SDS/PAGE and poly(vinylidene fluoride) transfer. Immunoblotting was performed with the following antibodies: rabbit anti‐STAT2 (ab233177, 1 : 1000), rabbit anti‐STAT2 (phosphor‐Y690; ab205718, 1 : 1000), rabbit anti‐IL‐2 (ab180780, 1 : 1000), rabbit anti‐GAPDH (1 : 1000), and goat anti‐(rabbit horseradish peroxidase) (ab6721, 1 : 5000; Abcam, Cambridge, UK). The blots were visualized with Enhance Chemiluminescence Kit (Millipore) according to the manufacturer’s instructions.

### Statistical analysis

All experiments were biologically repeated at least three times. All values were presented here as means ± standard deviation (SD). Differences between groups were evaluated by Student’s *t*‐test or one‐way ANOVA with a Tukey’s post hoc test. *P* values <0.05 were considered statistically significant.

## Results

### Knockdown of TRIM66 inhibited prostate cancer cell proliferation

**Figure 1:**
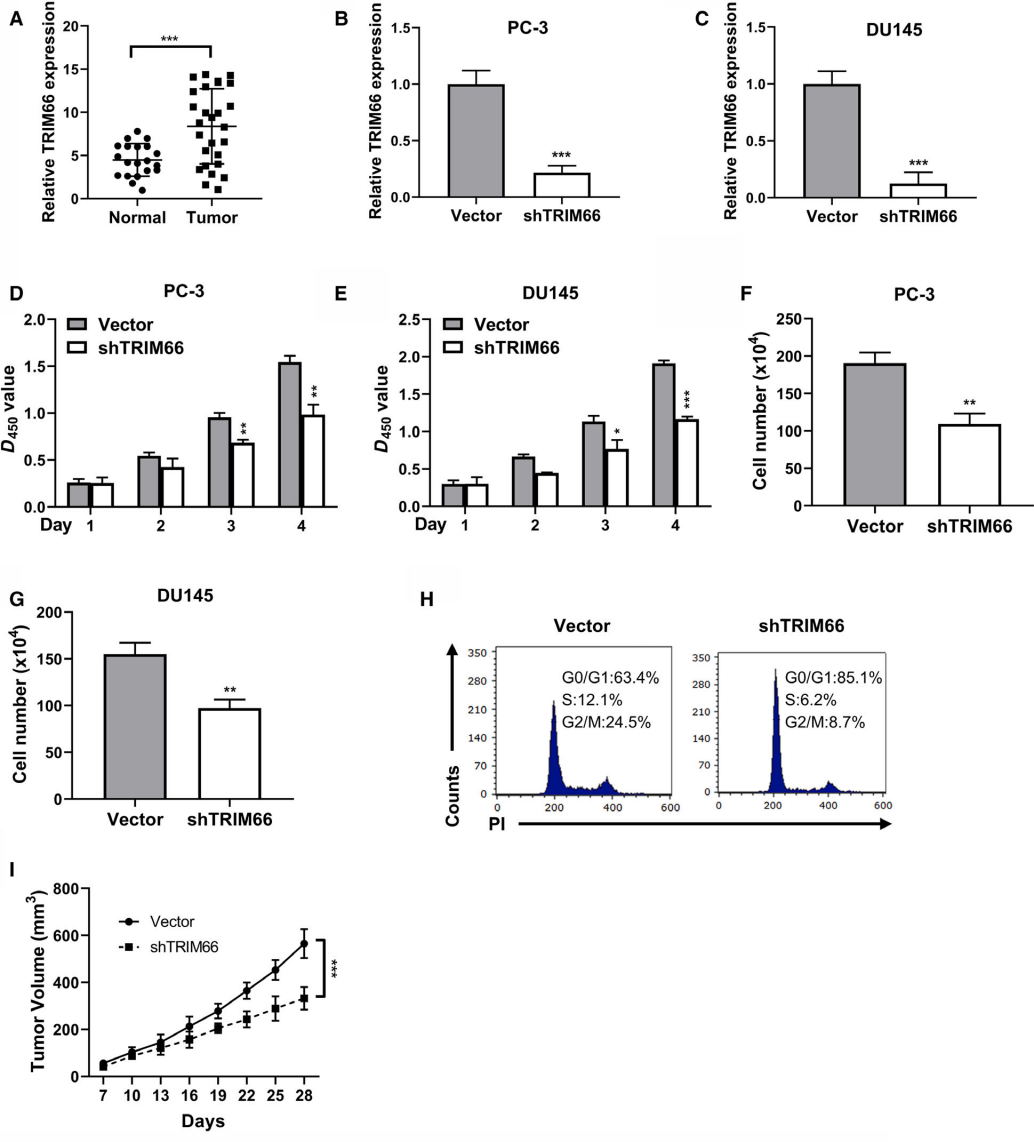
Knockdown of TRIM66 inhibited prostate cancer cells proliferation. (A) The expression of TRIM66 in prostate tumor tissues and the adjacent normal tissues was confirmed by quantitative reverse transcription PCR (RT‐qPCR). (B, C) The expression of TRIM66 in PC‐3 or DU145 cells transfected with shTRIM66 and corresponding vector were confirmed by RT‐qPCR. (D, E) Effects of TRIM66 depletion on the proliferation of PC‐3 and DU145 cells were detected by CCK‐8 assay. (F, G) Cell viabilities of PC‐3 and DU145 cells transfected with shTRIM66 and corresponding vector were determined by cell count assay. (H) Fluorescence‐activated cell sorting analysis of the effects of TRIM66 depletion on the cell cycle in PC‐3 cells. (I) Tumor growth curve of the PC‐3 cells transfected with vector or shTRIM66. All of the experiments were biologically repeated at least three times. Data were expressed as mean ± SD. (A–G) Data were analyzed by Student’s *t*‐test; (H) data were analyzed by one‐way ANOVA. *P < 0.05, **P < 0.01, ***P < 0.001. PI, propidine iodide.

In view of recognized oncogenic properties of TRIM66 in a number of human cancers, here we initially analyzed relative abundance of TRIM66 in clinical prostate tumor samples and found significant up‐regulation of TRIM66 in prostate cancer in comparison with normal control (Fig. 1A). We then established TRIM66‐deficient cells derived from two independent prostate cancer cell lines, PC‐3 and DU145. The success in establishment was confirmed by real‐time PCR, and more than 70% knockdown efficiency was achieved in both cell lines (Fig. 1B,C). Next, potential effects of TRIM66 deficiency on cell viability and proliferation were determined by CCK‐8 and direct cell counting, respectively. As shown in Fig. 1D,E, significant discrepancy was observed between control and TRIM66‐silenced cell lines at days 3 and 4 post‐inoculation. Cell propagation was greatly compromised by TRIM66 silencing in PC‐3 and DU145 cells, as well as indicated by the cell counting results (Fig. 1F,G). Moreover, TRIM66 depletion in PC‐3 cells caused cell‐cycle arrest at G0/G1 phase (Fig. 1H). In addition, PC‐3 cells transfected with vector or shTRIM66 were also inoculated into experimental mice, and the growth of the formed xenograft tumors was monitored, where tumors formed from PC‐3 cells with shTRIM66 were significantly slower than that with vector control (Fig. 1I). Therefore, our data suggested the oncogenic properties of TRIM66 in prostate cancer cells and TRIM66 knockdown greatly suppressed cell malignant proliferation *in vitro*.

### Knockdown of TRIM66 inhibited migration and invasion of prostate cancer cells

**Figure 2:**
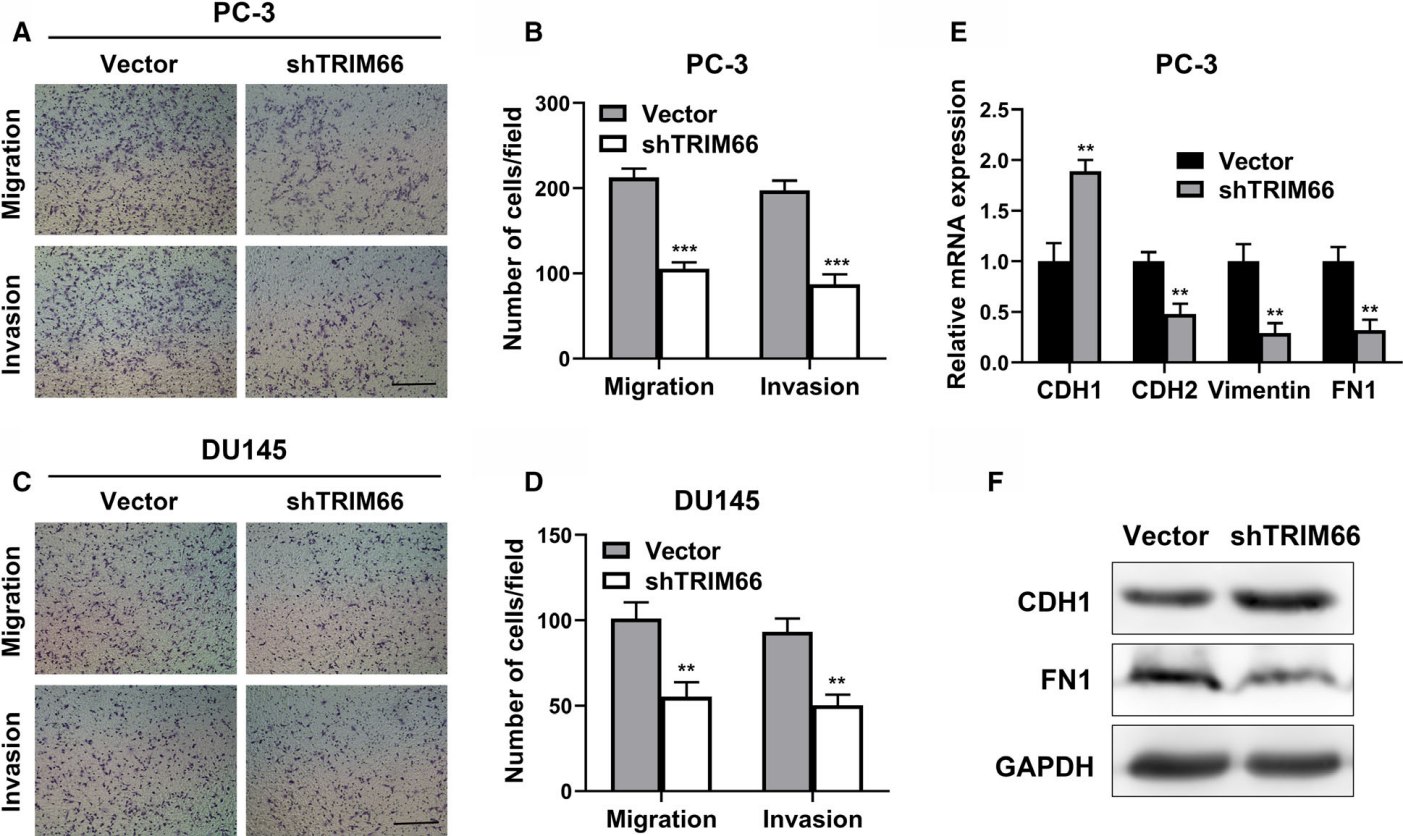
Knockdown of TRIM66 inhibited migration and invasion of prostate cancer cells. (A) The transwell of migration and invasion assay of PC‐3 cells transfected with shTRIM66 and corresponding vector. Scale bar, 200 μm. (B) The statistical results of the transwell assay in (A). (C) The transwell of migration and invasion of DU145 cells transfected with shTRIM66 and corresponding vector. Scale bar, 200 μm. (D) The statistical results of the transwell assay in (C). (E) The expression of EMT markers in PC‐3 cells transfected with shTRIM66 and corresponding vector was determined by RT‐qPCR. (F) The expression of EMT markers in PC‐3 cells transfected with shTRIM66 and corresponding vector was determined by western blot. All of the experiments were biologically repeated at least three times. Data were expressed as mean ± SD and analyzed by Student’s *t*‐test. **P < 0.01, ***P < 0.001.

Next, we sought to clarify the potential influence of TRIM66 silencing on the migrative and invasive capacities of prostate cancer cells. Transwell assay was used to evaluate the invasion and migration behaviors on TRIM66 knockdown. As shown in Fig. 2A–D, shRNA‐mediated knockdown of TRIM66 significantly decreased the migrated and invaded cell numbers in comparison with negative control in both cell lines, which suggested evidently compromised cell metastatic capacity by TRIM66 deficiency. Furthermore, we examined the EMT molecular markers, such as CDH1, CDH2, Vimentin and FN1. TRIM66 depletion remarkably stimulated the expression of epithelial marker CDH1 accompanied with simultaneous decreased expressions of mesenchymal markers CDH2, Vimentin and FN1 (Fig. 2E). We further validated our observation via examination of CDH1 and FN1 protein in both control and TRIM66‐knockdown PC‐3 cells (Fig. 2F). Our data clearly indicated that TRIM66 indispensably contributed to the EMT processing in prostate cancer cells.

### TRIM66 regulated STAT2 expression

**Figure 3:**
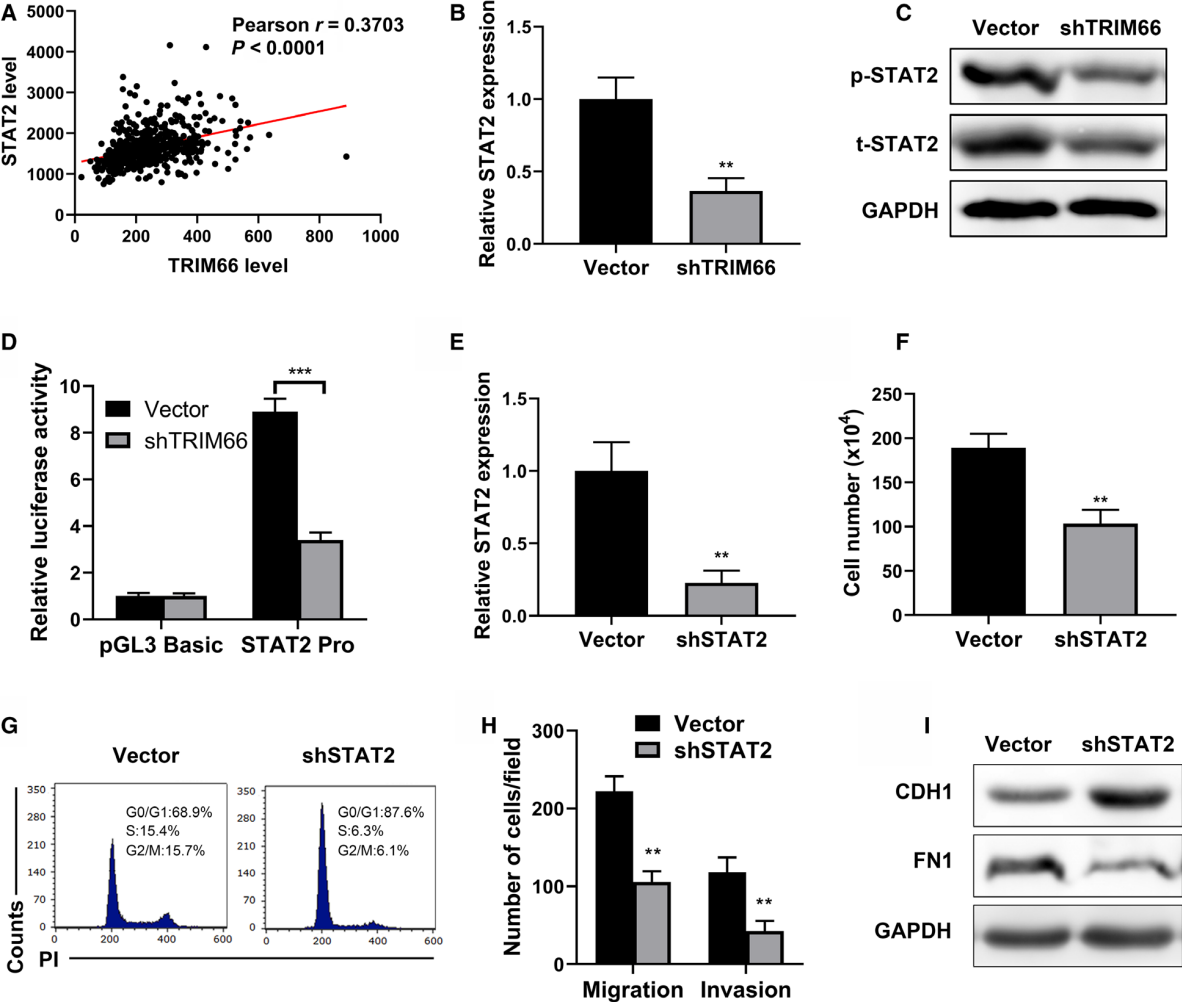
TRIM66 regulated STAT2 expression. (A) The correlation of TRIM66 and STAT2 in patients with prostate cancer was analyzed in TCGA prostate cancer dataset. (B) The mRNA expression of STAT2 in PC‐3 cells transfected with TRIM66 shRNA was determined by RT‐qPCR. (C) The protein expression of STAT2 and phosphorylated (p)‐STAT2 in PC‐3 cells transfected with TRIM66 shRNA was analyzed using western blot. (D) Luciferase reporter assay of STAT2 promoter in cells transfected with vector or shTRIM66. (E) The mRNA levels of STAT2 in PC‐3 cells transfected with shSTAT2 or corresponding vector were confirmed by RT‐qPCR. (F) Cell viabilities of PC‐3 cells transfected with shSTAT2 or corresponding vector were determined by cell count assay. (G) Fluorescence‐activated cell sorting analysis of the effects of STAT2 depletion on the cell cycle in PC‐3 cells. (H) Transwell of migration and invasion assay of PC‐3 cells transfected with shSTAT2 and corresponding vector. (I) The expression of EMT markers in PC‐3 cells transfected with shSTAT2 and corresponding vector was determined by western blot. All of the experiments were biologically repeated at least three times. Data were expressed as mean ± SD and analyzed by Student’s *t*‐test. **P < 0.01, ***P < 0.001.

Our previous study unambiguously underlined the importance of TRIM66 in prostate cancer cells with previously unacknowledged molecular mechanisms. In view of the assembly of evidences uncovering aberrances of JAK/STAT signaling in multiple forms of human cancers, here we focused on the potential linkage between TRIM66 and the JAK/STAT pathway in prostate cancer. To this end, we data‐mined The Cancer Genome Atlas (TCGA) database and retrieved the relative expression levels of TRIM66 and STAT2. A significant positive correlation between TRIM66 and STAT2 was observed (Fig. 3A, *P* < 0.0001). Endogenous STAT2 was tremendously suppressed at both transcript (Fig. 3B) and protein (Fig. 3C) levels in response to TRIM66 knockdown in PC‐3 cells. To simulate the down‐regulation of STAT2 in TRIM66‐deficient cells, we specifically silenced STAT2 in PC‐3 cells (Fig. 3D), and evidently inhibitory effects on cell proliferation, invasion and migration were noticed (Fig. 3E,3). We further demonstrated that STAT2 deficiency in PC‐3 cells led to cell‐cycle arrest at G0/G1 phase (Fig. 3G), accompanying decrease in both cell migration and invasion (Fig. 3H). EMT‐relevant molecular marker CDH1 was greatly induced, and FN1 was inhibited by STAT2 knockdown as well (Fig. 3I). The data acquired in STAT2‐deficient PC‐3 cells almost completely recapitulated the antitumor phenotypes elicited by TRIM66 knockdown, which implicated the important role of STAT2 in mediating the oncogenic activities of TRIM66 in prostate cancer cells.

### TRIM66 regulated IL‐2 expression

**Figure 4:**
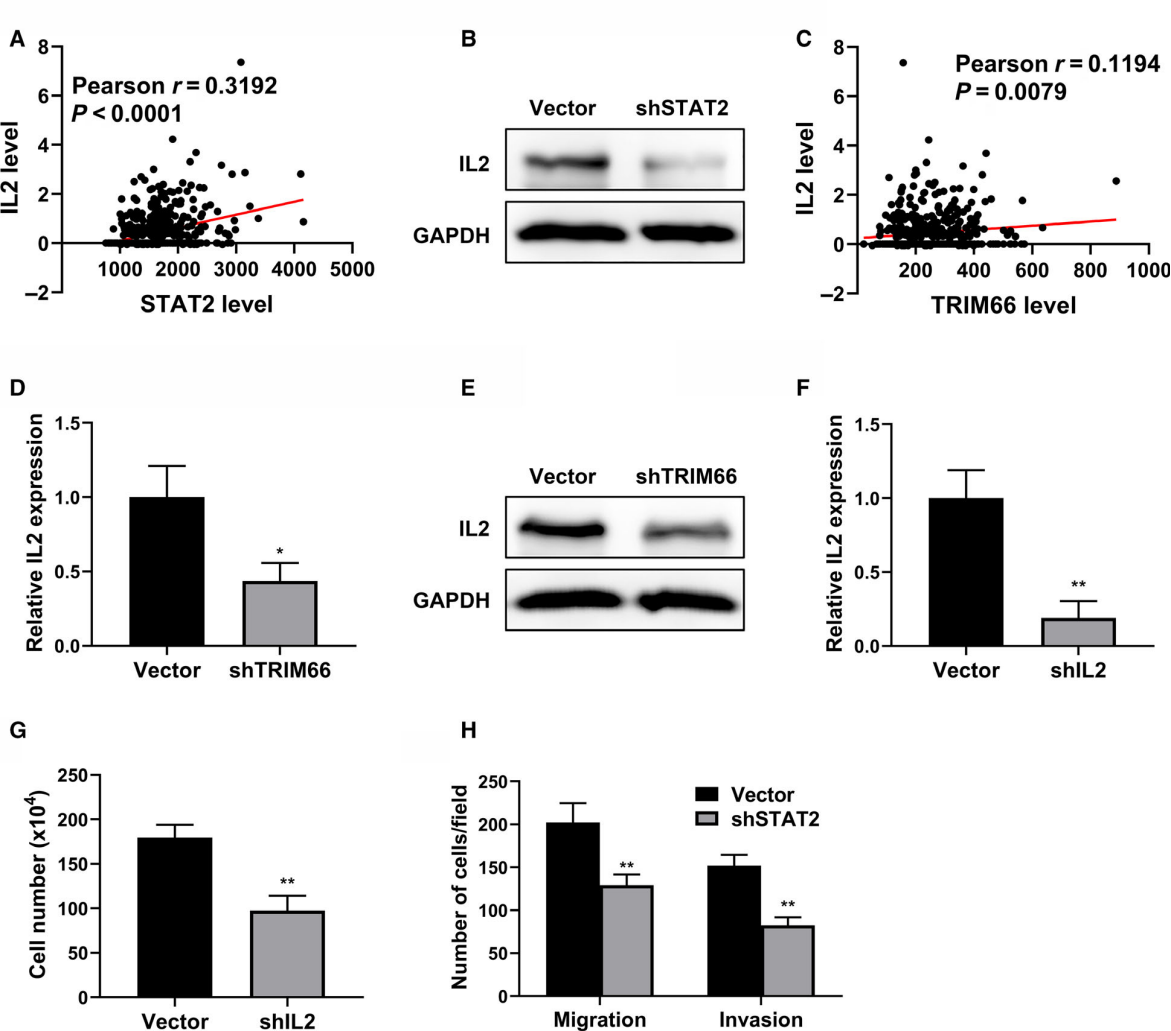
TRIM66 regulated IL‐2 expression. (A) The correlation of STAT2 and IL‐2 in patients with prostate cancer was analyzed in TCGA prostate cancer dataset. (B) The protein expression of IL‐2 in PC‐3 cells transfected with STAT2 shRNA was analyzed using western blot. (C) The correlation of TRIM66 and IL‐2 in patients with prostate cancer was analyzed in TCGA prostate cancer dataset. (D) The mRNA expression of IL‐2 in PC‐3 cells transfected with TRIM66 shRNA was determined by RT‐qPCR. (E) The protein expression of IL‐2 in PC‐3 cells transfected with TRIM66 shRNA was analyzed using western blot. (F) The mRNA levels of PC‐3 cells transfected with shIL‐2 or corresponding vector were confirmed by RT‐qPCR. (G) Cell viabilities of PC‐3 cells transfected with shIL‐2 or corresponding vector were determined by cell count assay. (H) Transwell of migration and invasion assay of PC‐3 cells transfected with shIL‐2 or corresponding vector. All of the experiments were biologically repeated at least three times. Data were expressed as mean ± SD and analyzed by Student’s *t*‐test. *P < 0.05, **P < 0.01.

In view of the complex regulatory network downstream of JAK/STAT signaling, we sought to further characterize the critical target molecule modulated indirectly by TRIM66 in prostate cancer cells. Gene expression correlation analysis disclosed a positive correlation between STAT2 and IL‐2 from TCGA database (Fig. 4A), which was validated in PC‐3 cells wherein STAT2 knockdown greatly inhibited IL‐2 expression (Fig. 4B). Again, a slightly but significantly positive correlation between TRIM66 and IL‐2 was observed in the clinical prostate tumor database (Fig. 4C, *P* < 0.01), which was consolidated by the down‐regulation of IL‐2 at both transcript (Fig. 4D) and protein (Fig. 4E) levels in TRIM66‐deficient PC‐3 cells. Next, we directly generated IL‐2‐silenced cells with shRNA (Fig. 4F), and effects similar to TRIM66 knockdown were demonstrated on proliferation, migration and invasion of PC‐3 cells (Fig. 4G,H). Our data suggested that IL‐2 was subjected to TRIM66/JAK/STAT regulation, which eventually contributed to the malignant growth and metastasis of prostate cancer cells.

### TRIM66 regulated prostate cancer cell proliferation and metastasis through mediating the STAT2–IL‐2 axis

**Figure 5:**
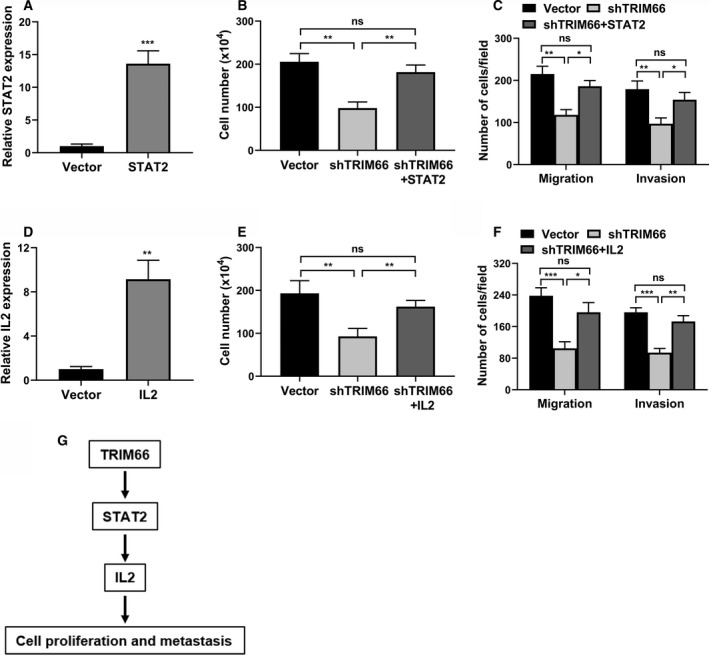
TRIM66 regulated prostate cancer cell proliferation and metastasis through mediating the STAT2–IL‐2 axis. (A) The mRNA expression of STAT2 in PC‐3 cells transfected with STAT2 overexpression plasmid was determined by RT‐qPCR. (B) Cell viability of PC‐3 cells transfected with shTRIM66 and/or STAT2 overexpression plasmid was determined by cell counts assay. (C) Transwell of migration and invasion assay of PC‐3 cells transfected with shTRIM66 and/or STAT2 overexpression plasmid. (D) The mRNA expression of IL‐2 in PC‐3 cells transfected with IL‐2 overexpression plasmid was determined by RT‐qPCR. (E) Cell viability of PC‐3 cells transfected with shTRIM66 and/or IL‐2 overexpression plasmid was determined by cell counts assay. (F) Transwell of migration and invasion assay of PC‐3 cells transfected with shTRIM66 and/or IL‐2 overexpression plasmid. (G) Schematic model of TRIM66‐mediated STAT2–IL‐2 signaling axis in prostate cancer cells. All of the experiments were biologically repeated at least three times. Data were expressed as mean ± SD. (A, D) Data were analyzed by Student’s *t*‐test, and others by one‐way ANOVA. *P < 0.05, **P < 0.01, ***P < 0.001.

Although our previous results showed that knockdown of both STAT2 and IL‐2 almost completely recapitulated the antitumor effects in response to TRIM66 depletion in prostate cancer cells, the predominance of STAT2–IL‐2 in mediating the oncogenic properties of TRIM66 in this disease was yet to be defined. To this purpose, we performed rescue assay to restore relative expression of either STAT2 or IL‐2 in TRIM66‐deficient PC‐3 cells, and addressed their impacts on cell proliferation, migration and invasion, respectively. Ectopic overexpression of STAT2 (Fig. 5A) significantly abolished the inhibitory actions on cell proliferation (Fig. 5B) and migration/invasion (Fig. 5C) imposed by TRIM66 knockdown. Although in IL‐2‐proficient PC‐3 cells (Fig. 5D), similar restorations in terms of cell proliferation, migration and invasion were observed in comparison with TRIM66 knockdown alone (Fig. 5E,5). Our data underlined the importance of STAT2–IL‐2 in mediating the protumor activities of TRIM66, and the signaling pathway elucidated was illustrated in Fig. 5G.

## Discussion

Despite the well‐recognized roles of TRIM66 in an array of human cancers, the expression status and mechanistic involvement of TRIM66 in prostate cancer were still elusive. Here we initiated the establishment of TRIM66‐silenced prostate cancer cells and determined their influence on malignant behaviors, such as cell viability, proliferation, migration and invasion. Our data uncovered that TRIM66 knockdown greatly compromised the cell viability and suppressed cell proliferation. In addition, the migrative and invasive capacities indicated by transwell assay were tremendously suppressed by TRIM66 depletion. Via data mining TCGA database, we noticed a positive correlation between TRIM66 and the JAK/STAT signaling pathway, especially STAT2 expression. The correlation was further validated in cell culture, wherein TRIM66 knockdown markedly decreased STAT2 expression at both transcript and protein levels. Consistently, STAT2 knockdown remarkably suppressed cell proliferation, invasion and migration. We further identified IL‐2 as the target gene downstream of STAT2, which manifested slight but significant positive correlations with both TRIM66 and STAT2, and was subjected to down‐regulation by both STAT2 and TRIM66. Similarly, we observed tremendous decreases in both the cell proliferative and migrative/invasive index in IL‐2‐deficient PC‐3 cells. To determine the predominance of STAT2–IL‐2 signaling in mediating the oncogenic properties of TRIM66 in prostate cancer cells, we performed rescue assay to force overexpressing either STAT2 or IL‐2 in TRIM66‐deficient PC‐3 cells, wherein both manipulations almost completely abolished the inhibitory effects by TRIM66 knockdown on cell proliferation, migration and invasion. Therefore, our study was clearly in support of the protumor activities of TRIM66 in prostate cancer cells, and corresponding mechanistic investigation highlighted the importance of the TRIM66–STAT2–IL‐2 axis underlying this malignant phenotype.

JAK/STAT signaling was one of the most common convergent downstream arrays of cytokine cues and was widely involved in cell proliferation, differentiation, apoptosis and inflammation processing 12, 13, 14. JAK/STAT signaling was also subjected to negatively regulatory loops and posttranslationally covalent modifications, which eventually formed complicated crosstalk with other pathways. Aberrant activation of the JAK/STAT pathway was characterized to play fundamental roles in the etiology and progression of multiple human diseases, including a number of cancers; it was under intensive investigation and development for its potential as a therapeutic target. Ruan *et al.* 15 reported that OCT4 accelerated initiation of transformation of ovarian cancer side population cells via activation of the JAK/STAT pathway. Khanna *et al.* 16 suggested that GRAMD1B modulated cell migration in breast cancer via both JAK/STAT and Akt pathways. In leukemia, Bailetti *et al.* 17 proposed that enhancer of Polycomb and Tip60 complex suppressed tumorigenesis through negatively modulating JAK/STAT signaling activation. Consistent with the recognized oncogenic properties of JAK/STAT signaling, here we demonstrated that this pathway was abnormally activated by TRIM66 in prostate cancer cells. Most importantly, we provided experimental evidence that shRNA‐mediated knockdown of STAT2 significantly compromised cell malignant behaviors. Our findings were also consolidated by a number of previous observations. For instance, Patel *et al.* 18 showed that ruxolitinib inhibited JAK/STAT signaling, and thus enhanced oncolytic virotherapy in non‐small cell lung cancer models. Xu *et al.* 19 suggested that inhibition of *BMX‐ARHGAP* fusion gene suppressed EMT in gastric cancer cells through RhoA‐mediated blockade of the JAK/STAT axis. Huang *et al.* proved in retinoblastoma cell lines that curcumin treatment compromised cell proliferation, invasion and migration, and stimulated apoptosis via regulation of miR‐99a and JAK/STAT signaling 20.

Notably, our results further uncovered the oncogenic activities of IL‐2 in prostate cancer cells, wherein IL‐2 was subjected to TRIM66‐positive regulation, and IL‐2 silencing greatly compromised cell proliferation, invasion and migration. Essentially as an IL, numerous investigations have disclosed the critical involvements of IL‐2 in antitumor immunity in multiple human malignancies. For instance, Jounaidi *et al.* 21 reported that tethering IL‐2 to its receptor significantly enhanced the antitumor and self‐expansion of natural killer NK92 cells. Sultan *et al.* 22 demonstrated that sustained persistence of IL‐2 cues promoted the antitumor properties of peptide vaccines via T‐cell expansion and releasing programmed cell death 1 (PD‐1) blockade. Menssen *et al.* 23 showed that both tumor necrosis factor‐α and IL‐2, delivered by the antibody‐based method to the blood vessel in solid tumor, manifested potently immunological and antitumor effects in the syngeneic J558L BALB/c myeloma model. Our data uncovered the novel role of IL‐2 in prostate cancer cells, and its knockdown evidently exerted antitumor action, which positioned itself as a potential target for therapeutic interventions.

Notably, despite the uncovered TRIM66–STAT2–IL‐2 prostate cancer, the molecular mechanisms underlying TRIM66‐mediated STAT2 regulation were still obscure, which warrants further investigations.

## Conclusions

In summary, here we have demonstrated the oncogenic role of TRIM66 in prostate cancer cells via positive regulation of JAK/STAT signaling, which holds great promise for therapeutic exploitations.

## Conflict of interest

The authors declare no conflict of interest.

## Author contributions

HC, RG, LC and YF conceived and designed the experiments. HC and RG performed the experiments. HC and RG analyzed and interpreted the data, and contributed reagents, materials, analysis tools or data. LC and YF wrote the paper.
